# Differentiation between fetal and postnatal iron deficiency in altering brain substrates of cognitive control in pre-adolescence

**DOI:** 10.1186/s12916-023-02850-6

**Published:** 2023-05-04

**Authors:** Mengdi Hua, Donglin Shi, Wenwen Xu, Liuyan Zhu, Xiaoxin Hao, Bingquan Zhu, Qiang Shu, Betsy Lozoff, Fengji Geng, Jie Shao

**Affiliations:** 1 Department of Child Health Care, Children’s Hospital, Zhejiang University School of Medicine, Hangzhou, China; 2 Department of Curriculum and Learning Sciences, Zhejiang University, Hangzhou, China; 3 National Clinical Research Center for Child Health, Hangzhou, China; 4 Department of Pediatrics, University of Michigan, Ann Arbor, MI USA

**Keywords:** Iron deficiency, Fetal, Postnatal, Cognitive control, Pre-adolescent children, Brain substrates

## Abstract

**Background:**

Early iron deficiency (ID) is a common risk factor for poorer neurodevelopment, limiting children’s potential and contributing to global burden. However, it is unclear how early ID alters the substrate of brain functions supporting high-order cognitive abilities and whether the timing of early ID matters in terms of long-term brain development. This study aimed to examine the effects of ID during fetal or early postnatal periods on brain activities supporting proactive and reactive cognitive control in pre-adolescent children.

**Methods:**

Participants were part of a longitudinal cohort enrolled at birth in southeastern China between December 2008 and November 2011. Between July 2019 and October 2021, 115 children aged 8–11 years were invited to participate in this neuroimaging study. Final analyses included 71 children: 20 with fetal ID, 24 with ID at 9 months (postnatal ID), and 27 iron-sufficient at birth and 9 months. Participants performed a computer-based behavioral task in a Magnetic Resonance Imaging scanner to measure proactive and reactive cognitive control. Outcome measures included accuracy, reaction times, and brain activity. Linear mixed modeling and the 3dlme command in Analysis of Functional NeuroImages (AFNI) were separately used to analyze behavioral performance and neuroimaging data.

**Results:**

Faster responses in proactive vs. reactive conditions indicated that all groups could use proactive or reactive cognitive control according to contextual demands. However, the fetal ID group was lower in general accuracy than the other 2 groups. Per the demands of cues and targets, the iron-sufficient group showed greater activation of wide brain regions in proactive vs. reactive conditions. In contrast, such condition differences were reversed in the postnatal ID group. Condition differences in brain activation, shown in postnatal ID and iron-sufficient groups, were not found in the fetal ID group. This group specifically showed greater activation of brain regions in the reward pathway in proactive vs. reactive conditions.

**Conclusions:**

Early ID was associated with altered brain functions supporting proactive and reactive cognitive control in childhood. Alterations differed between fetal and postnatal ID groups. The findings imply that iron supplement alone is insufficient to prevent persisting brain alterations associated with early ID. Intervention strategies in addition to the iron supplement should consider ID timing.

**Supplementary Information:**

The online version contains supplementary material available at 10.1186/s12916-023-02850-6.

## Background

Anemia, mostly caused by iron deficiency (ID), is prevalent worldwide [1, 2]. ID is still a leading risk factor for those aged 0–24 years, contributing to global burden [3], particularly in developing countries [1, 2]. Iron is essential for normal brain development, especially during fetal and early postnatal life, which are critical/sensitive periods of brain development [4–6]. Animal studies indicate that early ID alters brain structures and functions related to synaptogenesis, synaptic plasticity, myelination, and neurotransmitters, particularly in hippocampal and striatal/basal ganglia areas [7–10]. In humans, electrophysiological and behavioral studies suggest that even with iron treatment or supplementation, early ID is associated with long-lasting impairments in the development of attention, memory, and motor skills based on prefrontal, hippocampal, or sensorimotor brain regions [4, 11–14]. Such neurocognitive developmental processes underlie the late onset and maturation of high-order cognitive functions [15].

To understand developmental adaptation and neuroplasticity associated with early ID, it is important to investigate neural mechanisms underlying the long-term effects on the development of high-order cognitive functions. To date, only two studies used functional magnetic resonance imaging to examine long-term effects of early ID on human brain functions. The results showed that ID anemia in infancy altered the co-activation of brain regions during the resting state in adulthood [16, 17]. Although these two preliminary studies are ground-breaking, there are limitations. First, information on iron status at birth was not available, meaning that the two studies could not consider the timing of early ID. Additionally, these two studies measured brain functions during the resting state, which lacks specificity in terms of high-order cognitive functions. Finally, Since magnetic resonance imaging methodologies were not feasible in the study context until participants were young adults, the study also could not offer insight into ID-associated changes in childhood or adolescence — which are sensitive periods for the development of high-order cognitive functions, such as cognitive control [18]. To fill these gaps, we applied task-based functional Magnetic Resonance Imaging methodologies in our longitudinal cohort to examine brain mechanisms underlying the long-term effects of fetal ID and postnatal ID on cognitive control in pre-adolescent children.

Alteration in cognitive control is associated with many developmental disorders (e.g., attention deficit hyperactivity disorder) [19]. Cognitive control — a high-order cognitive function — refers to the regulation of behaviors and thoughts in accordance with internally maintained goals [20]. According to the dual mechanisms of the control framework, cognitive control operates via two distinct operating modes, proactive control and reactive control [21]. The proactive control promotes the configuration of neurocognitive systems in advance according to internally maintained goals, whereas the reactive control refers to the stimulus-triggered just-in-time activation of brain systems to facilitate task processing [21]. As children get older, they gradually transition from mainly using reactive control to recruiting proactive control flexibly [22–24]. The flexible use of cognitive control depends on multiple brain networks (e.g., fronto-parietal and midcingulo-opercular networks) [25, 26] that do not reach maturity at least until late adolescence [27]. The long-term development of these brain networks is based on the brain circuits formed in early life [27]. As early brain development requires adequate iron supply [4], early ID may exert detrimental influences on brain development. Such influences may vary depending on early ID timing as the brain develops very rapidly in early life [28–30]. Therefore, we predicted that fetal ID and postnatal ID would both reshape brain development but in different ways, contributing to long-lasting alterations of brain functions that support the flexible use of cognitive control in pre-adolescent children.

To test the prediction, we recalled participants aged 8–11 years from a birth cohort established in southeastern China with documented iron status since birth. Then, we used task-based functional magnetic resonance imaging to collect brain activity data while participants were performing the cognitive control task in the scanner. This task includes proactive and reactive conditions. Compared to the non-informative cues in the reactive condition, the informative cues in the proactive condition can induce proactive control, enabling the recruitment of brain resources to select response rules before the onset of targets. Such selection in advance can support faster and more accurate responses to targets. We hypothesized that there were differences between the fetal ID, postnatal ID, and control groups in recruiting brain resources to support the cognitive processing in proactive vs. reactive conditions.

## Methods

### Participants

Participants were recruited by the Brain and Behavior in Early Iron Deficiency study, a collaboration between the University of Michigan and the Children’s Hospital, Zhejiang University School of Medicine. This was a longitudinal cohort project that aimed to evaluate the impact of early ID on neurodevelopment. Healthy full-term infants were enrolled at birth between December 2008 and November 2011 at the Fuyang Maternal and Children’s Health Care Hospital in southeastern China [31]. The infants enrolled in the Brain and Behavior in Early Iron Deficiency study met the following entrance criteria [31]: singleton term, birth weight ≥ 2500 g, 5-min Apgar score ≥ 7, and no delivery complications, maternal or infant health problems, or major congenital deficits. This study was approved by the Institutional Review Boards of the University of Michigan and the Children’s Hospital, Zhejiang University School of Medicine. The demographic information, such as parental age, parental education, parental occupation, and household income, was collected by qualified project personnel when the cohort study was set up (i.e., at 6-week-old and 9-month-old). The family socioeconomic status was assessed by the combination of parental education, parental occupation, and household income (Additional file 1: The computation of family socioeconomic status).

Between October 2019 and October 2021, 115 children aged 8–11 years and their families were invited back to participate in this study. Nineteen children declined to go into the scanner or did not finish the test. Another 25 children were excluded for the following reasons: technical problems (2), low behavioral performance (2), health reasons (1), or too much head motion [20]. After the attrition, 71 participants were included in analyses (mean age = 10.0 years, SD = 0.9, 37 males, 34 females). This Zhejiang University follow-up study was approved by the Institutional Review Board of the Children’s Hospital, Zhejiang University School of Medicine. Before participating in this study, parents and children separately signed consent and assent forms.

### Iron status

Iron status was assessed at birth using cord blood and repeated at 9 months, 18 months, and 8–11 years old using venous blood. Measures of iron status included serum ferritin (SF), zinc protoporphyrin/heme ratio (ZPP/H), mean corpuscular volume (MCV), and/or red cell distribution width (RDW). Hemoglobin (Hb) was used to assess anemia status. Serum C-reactive protein was measured to exclude inflammation that may interfere with the diagnosis of iron status. The iron assay methods were documented in our previous publications [30–32]. Fetal ID was defined as cord SF < 75 µg/l or ZPP/H > 118 µmol/mol [33–35]; postnatal ID was defined as normal cord iron status at birth and ≥ 2 abnormal iron measures at 9 months using the following cutoffs: MCV < 74 fl [36], RDW > 14.5% [37], SF < 12.0 µg/l [38], and ZPP/H > 69 µmol/mol [39]; iron sufficiency refers to no ID both at birth and 9 months. Infants with cord SF < 60 µg/L randomly received the iron supplement or placebo according to the design of a small randomized controlled trial [32]. All children with ID anemia at 9 months received iron therapy. In the sample for functional magnetic resonance imaging analyses, there were 20 children with fetal ID, 24 children with postnatal ID, and 27 children with iron sufficiency in early life. Among children with fetal ID, 8 children had ID only at birth, and 12 children had ID both at birth and 9 months. No participant had ID or ID anemia at 18 months and 8–11 years. The 3 groups did not differ in sex, age, head motion, parental age, parental education, parental occupation, household income, and family socioeconomic status (*ps* > 0.10). More details are provided in Additional file 1.

### Behavioral task

**Fig. 1:**
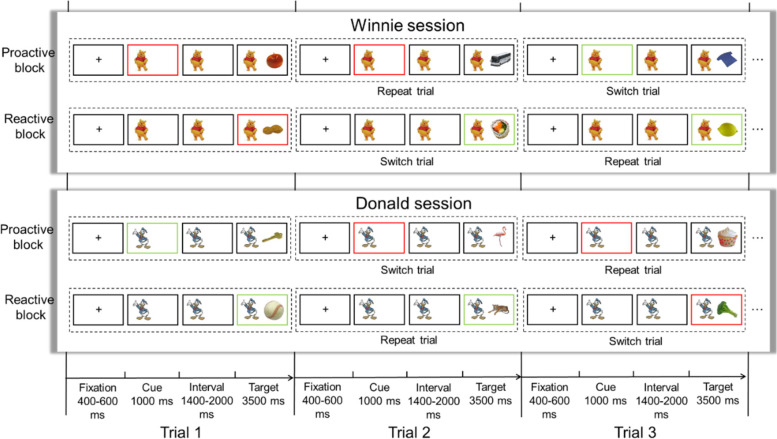
Design of the cued task-switching paradigm. To counterbalance stimuli positions and response rules across participants, we created 4 versions of this task. Each participant was randomly assigned to one version (one example version was presented above). In each version, there were Winnie and Donald sessions. In each session, there were 80 trials, and each trial included the periods of Fixation, Cue, Interval, and Target. In proactive blocks, the border was green or red during the Cue period, whereas such colored border was not presented until the presence of targets in the reactive block. Participants were asked to make responses in Target periods by pressing the left or right key in each trial. Response rules were jointly determined by the cartoon character and colored border and differed between Winnie and Donald sessions. For example, in the Winnie session, the green border indicated that the rule was to judge whether the target was a food picture (i.e., green-food rule), and the red border was to judge whether the target was an animal picture (i.e., red-animal rule). Compared to the Winnie session, rules in the Donald session were reversed (i.e., red-food and green-animal). Such task rules were counterbalanced across participants

Participants performed a widely used computer-based behavioral task [22, 40] in the Magnetic Resonance Imaging scanner to measure proactive and reactive cognitive control (Fig. 1, Additional file 1: Protocol of the behavioral task). It included 2 sessions with background cartoon characters of Winnie or Donald. In each session, 2 proactive blocks and 2 reactive blocks were administrated randomly. In proactive blocks, a colored border was presented with one cartoon character as an informative cue, followed by a target picture of food, animal, or objects. In reactive blocks, black borders were presented with cartoon characters as non-informative cues, and colored borders were not presented until the onset of target pictures. Colored borders and cartoon characters jointly determined response rules. According to such rules, participants were asked to respond to target pictures by pressing buttons using the left or right index finger. Thus, proactive blocks allowed participants to figure out the rule of a trial before the onset of targets, but the rule in reactive blocks could not be identified until the onset of targets. There were 20 target pictures in each block, which contained switch and repeat conditions and each condition had 8–11 trials. For switch trials, rules in current trials were changed compared to previous trials, whereas there was no such change in repeat trials. Block duration was 2 min and 13 s with a 17-s break between blocks. Stimuli positions and response rules were counterbalanced across participants.

### Imaging data acquisition and preprocessing

Participants were scanned in a Siemens 3.0-T scanner (MAGNETOM Prisma, Siemens Healthcare Erlangen, Germany) using a 60-channel coil. Before formal scanning, children learned how to stay still in a mock scanner. Then, participants completed a series of structural and functional scans in the real scanner. The structural images were acquired with a T1-weighted magnetization prepared rapid gradient echo sequence: TR (repetition time) = 2300 ms, TE (echo time) = 2.32 ms, slice thickness = 0.9 mm, voxel size = 0.90 × 0.90 × 0.90 mm^3^, voxel matrix = 256 × 256, flip angle = 8°, field of view = 240 mm. During task scanning, a total of 546 whole-brain volumes were collected using a T2-weighted gradient echo planar imaging sequence with multi-bands acceleration (TR = 1000 ms, TE = 34 ms, slice thickness = 2.50 mm, voxel size = 2.50 × 2.50 × 2.50 mm^3^, voxel matrix = 92 × 92, flip angle = 50°, field of view = 230 mm^2^, slice number = 52, MB-factor = 4).

Preprocessing neuroimaging data consisted of several steps. First, slice timing and head motion were corrected using AFNI (Analysis of Functional NeuroImages, Cox, 1996). Then, tissue segmentation was conducted to extract brains using SPM12 (Statistical Parametric Mapping 12, https://www.fil.ion.ucl.ac.uk/spm/). Structural and functional images were normalized to the MNI (Montreal Neurological Institute) space using ANTs (Advanced Normalization Tools, http://stnava.github.io/ANTs/). Finally, spatial smoothing was conducted with a 5-mm full-width-at-half-maximum Gaussian kernel. For first-level analyses, multiple regression analyses were conducted using the 3dDeconvolve command within AFNI. Events during encoding were convolved with the hemodynamic function to create a total of 8 regressors of interest (2 sessions: session 1 vs. 2; 2 control conditions: proactive vs. reactive; 2 switch conditions: repeat vs. switch) for cue- and target-elicited brain activity. Six head motion parameters were included as covariates. To alleviate the impact of head motion, we censored the volumes with framewise displacement > 0.5 mm. All subjects included for statistical analyses had mean framewise displacement (FD) within 0.13 to 0.44 mm.

### Statistical analyses

Linear mixed modeling (IBM SPSS Statistics 22, IBM Corp., Chicago, IL, USA) was used to analyze behavioral performance as indexed by reaction times and accuracy. In fixed-effect models, Group, Control, Switch, and their interactions were included as predictors. The subject factor was included in the random-effect model. We compared the models that contained only main effects to the ones that also included interactions. We selected the best-fitting models with the lowest Akaike’s Information Criterion (AIC) values. The selected models and their AIC values were presented in Additional file 1. For neuroimaging data, the 3dlme command in AFNI was used to test the main effects of Group, Control, Switch, and their interactions in predicting cue- and target-elicited brain activity. The 3dClustSim mixed model autocorrelation function indicated that clusters with uncorrected *p* < 0.001 and a minimum of 27 voxel size remained significant after multiple comparison corrections (*p*_corrected_ < 0.05). Such correction was carried out for all reported neuroimaging results. The results below focus on differences between groups in behavioral performance and brain activity.

## Results

### Behavioral performance

Children in all groups showed faster responses in proactive vs. reactive conditions (*β* =  − 428.0; 95% CI, − 483.5 to − 372.4; *p* < 0.001), but there was no significant difference in reaction times between groups. For accuracy, no group showed statistically significant differences between proactive and reactive conditions. The fetal ID group was lower in overall accuracy than both the postnatal ID (*β* =  − 0.06; 95% CI, − 0.10 to − 0.01; *p* = 0.02) and iron-sufficient groups (*β* =  − 0.08; 95% CI, − 0.12 to − 0.04; *p* = 0.001). More details are provided in Additional file 1: Table S4.

### Neuroimaging results

**Fig. 2:**
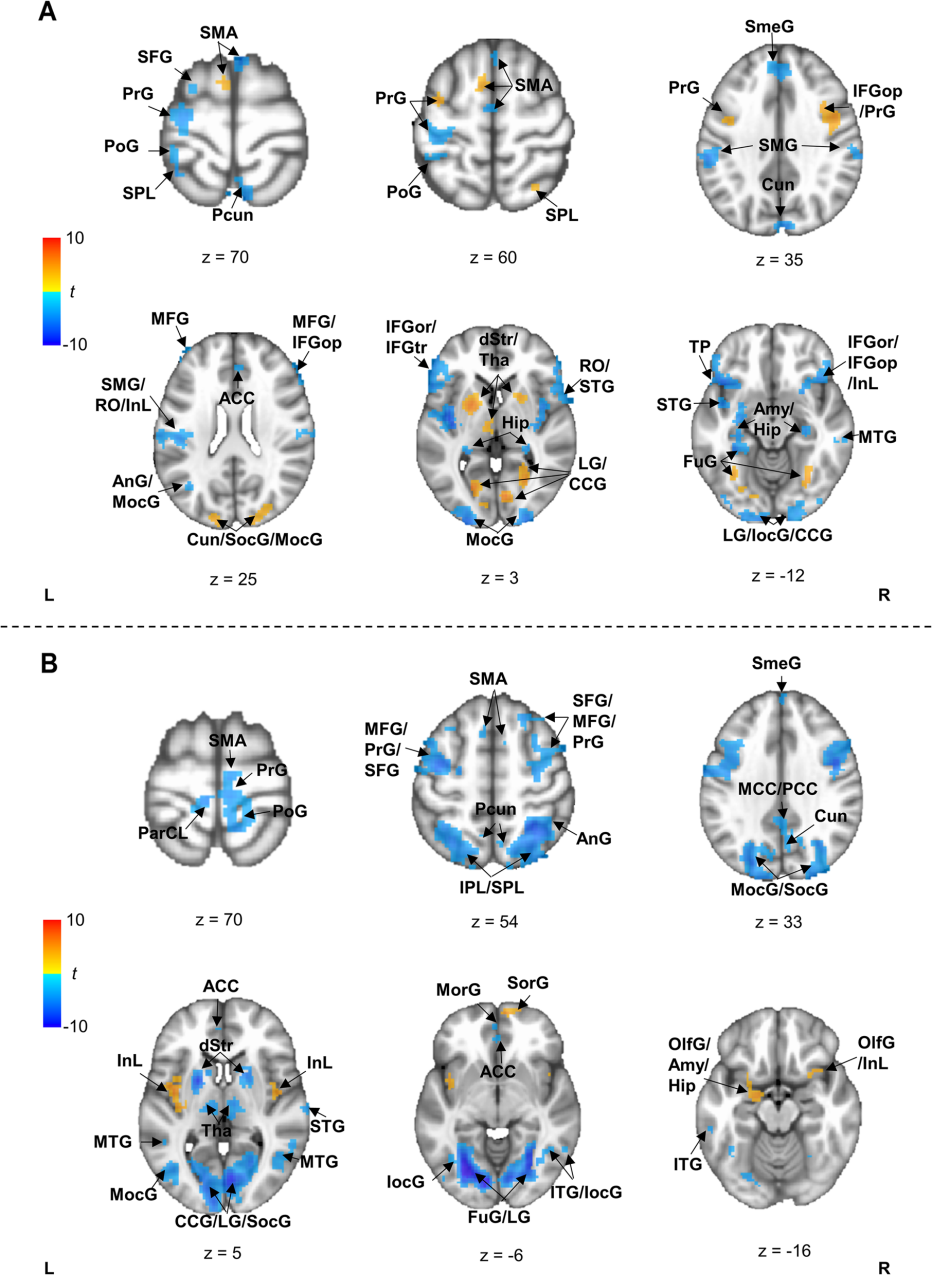
Common brain regions recruited by three groups to process cues (**A**) and targets (**B**) in proactive vs. reactive conditions. Orange patches denote common brain regions showing greater activation or weaker deactivation to process cues or targets in proactive vs. reactive conditions, while common brain regions showing reversed condition differences are represented by blue patches. The *t* values denote differences between proactive and reactive conditions. ACC, anterior cingulate cortex; Amy, amygdala; AnG, angular gyrus; CCG, calcarine gyrus; Cun, cuneus; dStr, dorsal striatum; FuG, fusiform gyrus; Hip, hippocampus; IFGor, inferior frontal gyrus (p. orbitalis); IFGop, inferior frontal gyrus (p. opercularis); IFGtr, inferior frontal gyrus (p. triangularis); InL, insula; IocG, inferior occipital gyrus; IPL, inferior parietal lobule; ITG, inferior temporal gyrus; LG, lingual gyrus; MCC, middle cingulate cortex; MFG, middle frontal gyrus; MocG, middle occipital gyrus; MorG, mid orbital gyrus; MTG, middle temporal gyrus; Hip, hippocampus; OlfC, olfactory cortex; ParCL, paracentral lobe; PCC, posterior cingulate cortex; PoG, postcentral gyrus; PrG, precentral gyrus; Pcun, precuneus; RO, rolandic operculum; SFG, superior frontal gyrus; SMA, supplemental motor area; SmeG, superior medial gyrus; SMG, supramarginal gyrus; SocG, superior occipital gyrus; SorG, superior orbital gyrus; SPL, superior parietal lobe; STG, superior temporal gyrus; Tha, thalamus; TP, temporal pole

**Fig. 3:**
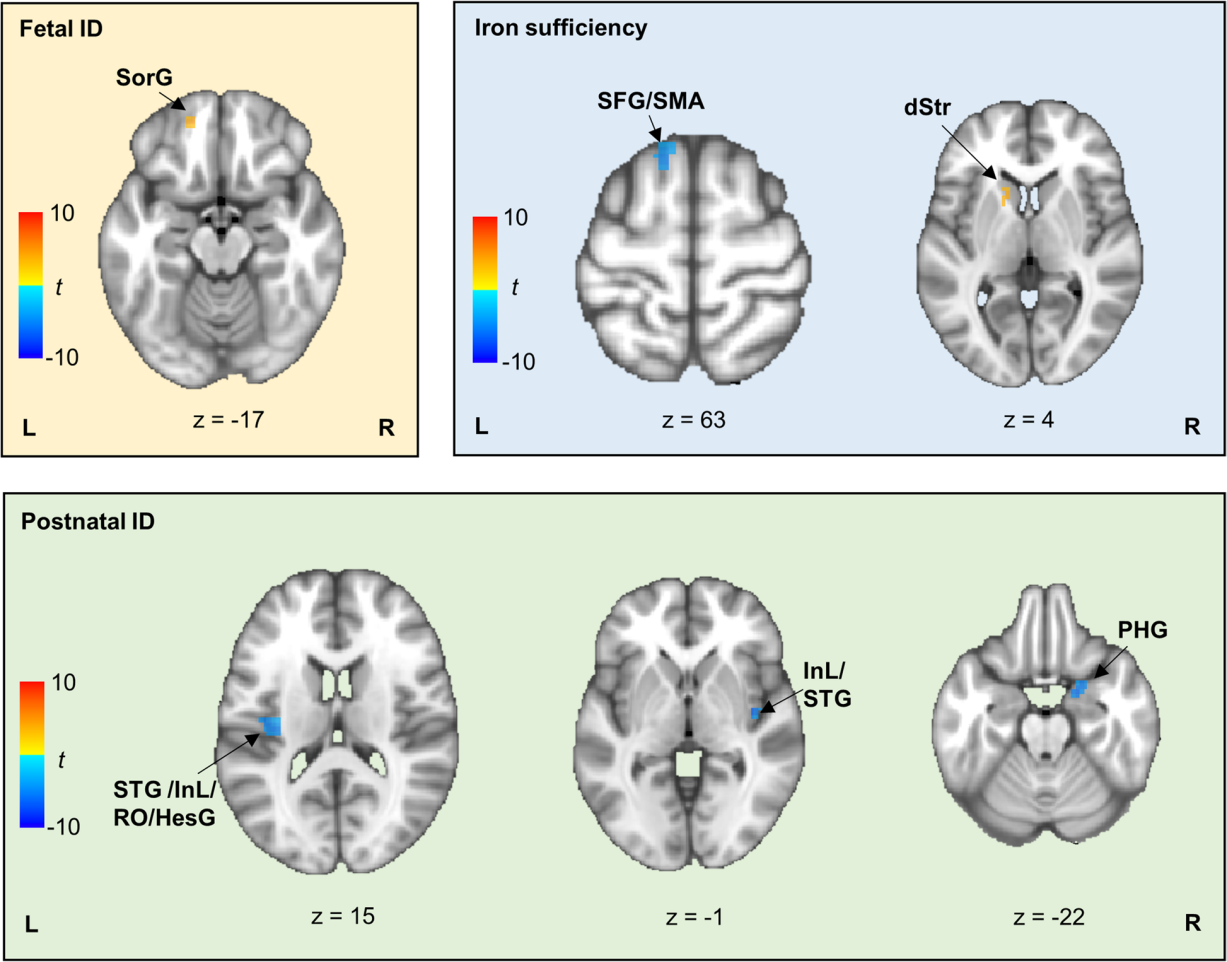
Specific brain regions recruited by each group to process cues in proactive vs. reactive conditions. In each group, orange patches denote regions showing greater activation or weaker deactivation to process cues in proactive vs. reactive conditions, while blue patches denote regions showing reversed conditional differences. The *t* values denote differences between proactive and reactive conditions. dStr, dorsal striatum; HesG, heschl’s gyrus; InL, insula; PHG, parahippocampus; RO, rolandic operculum; SFG, superior frontal gyrus; SMA, supplemental motor area; SorG, superior orbital gyrus; STG, superior temporal gyrus

**Fig. 4:**
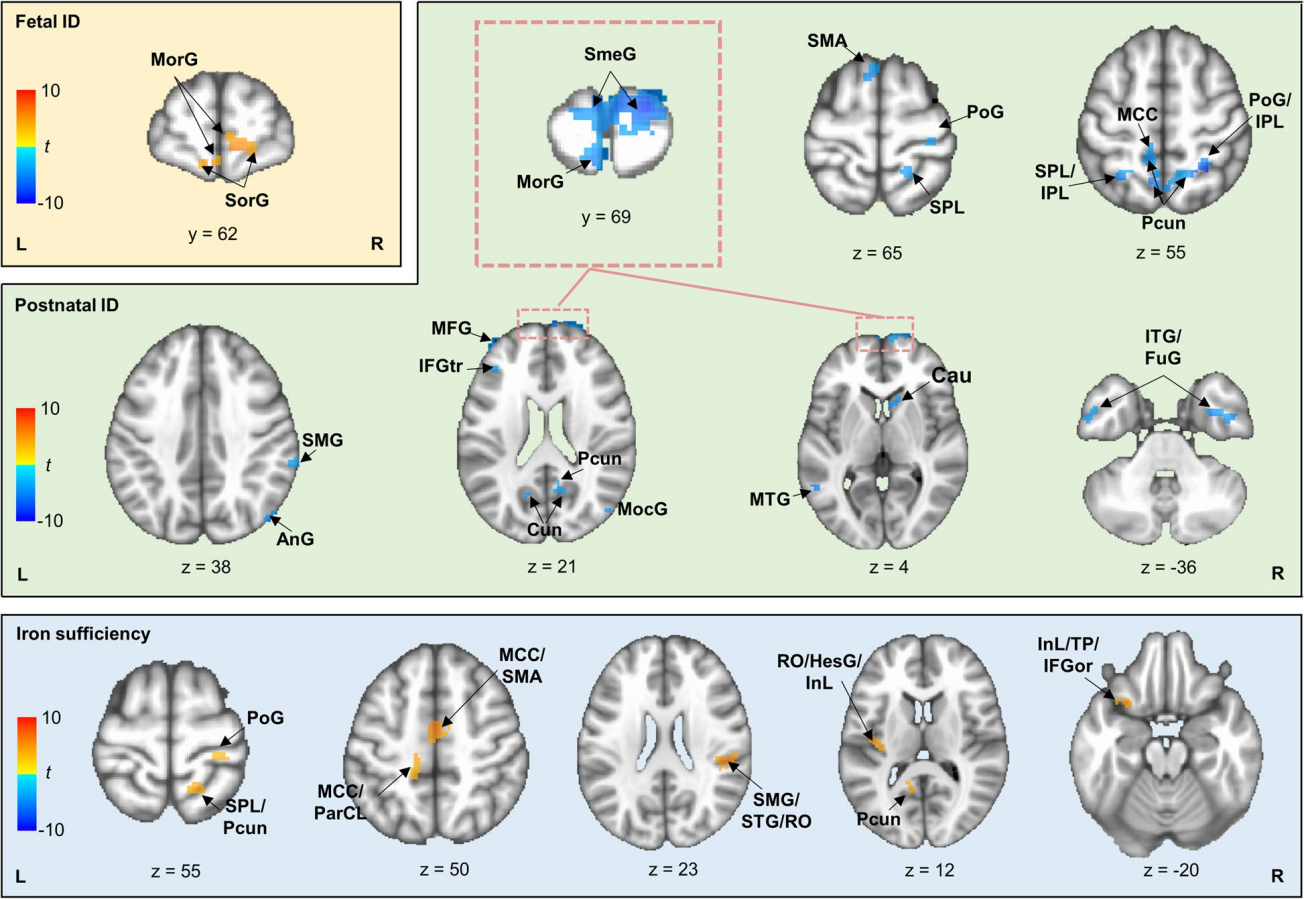
Specific brain regions recruited by each group to process targets in proactive vs. reactive conditions. In each group, orange patches denote regions showing greater activation or weaker deactivation to process targets in proactive vs. reactive conditions, while blue patches denote regions showing reversed conditional differences. The *t* values denote differences between proactive and reactive conditions. AnG, angular gyrus; Cau, caudate nucleus; Cun, cuneus; HesG, heschl’s gyrus; MCC: middle cingulate cortex; FuG, fusiform gyrus; MFG, middle frontal gyrus; MocG, middle occipital gyrus; MorG, mid orbital gyrus; MTG, middle temporal gyrus; IFGor, inferior frontal gyrus (p. orbitalis); IFGtr, inferior frontal gyrus (p. triangularis); InL, insula; IPL, inferior parietal lobe; ITG, inferior temporal gyrus; ParCL, paracentral lobe; Pcun, precuneus; PoG, postcentral gyrus; RO, rolandic operculum; SMA, supplemental motor area; SmeG, superior medial gyrus; SMG, supramarginal gyrus; SorG, superior orbital gyrus; STG, superior temporal gyrus; SPL, superior parietal lobe; TP, temporal pole

Consistent with behavioral results, all groups showed significant differences in cue- and target-elicited brain activity between proactive and reactive conditions (Fig. 2; Additional file 1: Table S5-S6). *Additionally*, such condition differences in brain activity varied between groups, as evidenced by significant Group × Control interactions at 6 clusters for cue-elicited brain activity (Table 1, Fig. 3) and at 21 clusters for target-elicited brain activity (Table 2, Fig. 4).

**Table 1:** Brain regions showing significant Control × Group interaction in cue-elicited activity

**Cluster**	**Hemi**	**Peak MNI coordinate**			**Size**	**Fetal ID**			**Postnatal ID**			**IS**		
		***x***	***y***	***z***		**Pro**	**Rea**	**DIF**	**Pro**	**Rea**	**DIF**	**Pro**	**Rea**	**DIF**
**Posterior rolandic operculum**	L	− 38	− 23	16	92	ns	ns	ns	ns	+	−	ns	ns	ns
*Posterior insula*	L					ns	ns	ns	ns	+	−	ns	ns	ns
*Heschel’s gyrus*	L					ns	ns	ns	ns	+	−	ns	ns	ns
*Superior temporal gyrus*	L					ns	ns	ns	ns	+	−	ns	ns	ns
**Posterior insula**	R	42	− 10	− 2	45	ns	ns	ns	ns	+	−	ns	ns	ns
*Superior temporal gyrus*	R					ns	ns	ns	ns	+	−	ns	ns	ns
**Superior frontal gyrus**	L	− 13	25	63	42	ns	ns	ns	ns	ns	ns	ns	+	−
*Supplemental motor area*	L					ns	ns	ns	ns	ns	ns	ns	+	−
**Caudate nucleus**	L	− 13	15	3	32	ns	ns	ns	ns	ns	ns	ns	−	+
*Putamen*	L					ns	−	+	ns	ns	ns	ns	−	+
**Parahippocampus**	R	22	5	− 22	31	ns	ns	ns	ns	+	−	ns	ns	ns
**Superior orbital gyrus**	L	− 15	50	− 17	29	+	ns	+	ns	ns	ns	ns	ns	ns

Among brain regions showing significant Control × Group interaction, we conducted further analyses to see whether there was significant condition difference in activity at these brain regions for each group. In Pro and Rea columns, “ + ” and “ − ” separately indicate activation and deactivation; in the DIF column, “ + ” indicates greater brain activation or weaker deactivation in proactive vs. reactive conditions and opposite differences are represented by “ − ”. Brain regions in bold are at the peak MNI coordinates of clusters. Other brain regions in these clusters are in italics

*ID* Iron deficiency, *IS* Iron sufficiency, *Hemi* Hemisphere, *R* Right, *L* Left, *Pro* Proactive, *Rea* Reactive, *DIF* Differences

**Table 2:** Brain regions showing significant Control × Group interaction in target-elicited activity

**Cluster**	**Hemi**	**Peak MNI coordinate**			**Size**	**Fetal ID**			**Postnatal ID**			**IS**		
		***x***	***y***	***z***		**Pro**	**Rea**	**DIF**	**Pro**	**Rea**	**DIF**	**Pro**	**Rea**	**DIF**
**Superior orbital gyrus**	B	15	65	− 4	295	ns	−	+	ns	ns	ns	ns	ns	ns
*Mid orbital gyrus*	B					ns	−	+	−	ns	−	ns	ns	ns
*Superior medial gyrus*	B					ns	ns	ns	−	ns	−	ns	ns	ns
**Superior parietal lobe**	R	15	− 58	58	290	ns	ns	ns	ns	+	−	+	ns	+
*Postcentral gyrus*	R					ns	ns	ns	ns	+	−	+	ns	+
*Posterior precuneus*	B					ns	ns	ns	ns	+	−	+	ns	+
**Middle cingulate cortex**	B	2	− 8	48	174	ns	ns	ns	ns	ns	ns	+	ns	+
*Supplementary motor area*	B					ns	ns	ns	+	+	ns	+	+	+
**Posterior middle cingulate cortex**	L	− 14	− 42	50	112	ns	ns	ns	−	ns	−	+	ns	+
*Paracentral lobe*	L					ns	ns	ns	ns	ns	ns	+	ns	+
*Anterior precuneus*	L					ns	ns	ns	−	ns	−	ns	ns	ns
**Posterior precuneus**	R	15	− 60	23	91	ns	ns	ns	−	ns	−	ns	ns	ns
*Cuneus*	L					ns	ns	ns	−	ns	−	ns	ns	ns
**Supramarginal gyrus**	R	50	− 35	31	87	ns	ns	ns	ns	ns	ns	+	ns	+
*Superior temporal gyrus*	R					ns	ns	ns	−	ns	−	+	ns	+
**Posterior precuneus**	L	− 8	− 58	13	86	ns	ns	ns	ns	ns	ns	+	ns	+
*Cuneus*	L					ns	ns	ns	ns	ns	ns	ns	−	+
**Posterior insula**	L	− 33	− 18	8	58	ns	ns	ns	ns	+	ns	+	ns	+
*Heschel’s gyrus*	L					ns	ns	ns	ns	ns	ns	+	ns	+
*Posterior rolandic operculum*	L					ns	ns	ns	ns	ns	ns	+	ns	+
**Anterior inferior temporal gyrus**	R	42	− 8	− 42	51	ns	ns	ns	ns	+	−	+	+	ns
*Anterior fusiform gyrus*	R					ns	ns	ns	ns	+	−	+	+	ns
**Postcentral gyrus**	R	30	− 30	68	51	ns	ns	ns	ns	ns	ns	+	ns	+
**Angular gyrus**	R	47	− 73	31	49	ns	ns	ns	ns	+	−	ns	ns	ns
*Middle occipital gyrus*	R					ns	ns	ns	ns	+	−	ns	ns	ns
*Middle temporal gyrus*	R					ns	ns	ns	ns	+	−	ns	ns	ns
**Postcentral gyrus**	L	− 20	− 50	56	49	ns	ns	ns	ns	+	−	ns	ns	ns
*Superior parietal lobe*	L					ns	+	ns	ns	+	−	ns	ns	ns
*Inferior parietal lobe*	L					ns	+	ns	ns	+	−	ns	ns	ns
**Anterior inferior temporal gyrus**	L	− 53	− 3	− 37	48	ns	ns	ns	ns	+	−	ns	ns	ns
**Middle frontal gyrus**	L	− 38	35	23	40	ns	ns	ns	ns	+	−	+	+	ns
*Inferior frontal gyrus (p. triangularis)*	L					ns	ns	ns	ns	+	−	+	+	ns
**Postcentral gyrus**	R	27	− 48	56	38	ns	+	ns	ns	+	−	ns	ns	ns
*Superior parietal lobe*	R					ns	+	ns	ns	+	−	ns	ns	ns
**Caudate nucleus**	R	12	17	3	37	ns	ns	ns	−	ns	−	ns	ns	ns
**Supplementary motor area**	L	− 5	15	66	35	ns	+	ns	ns	+	−	+	+	ns
**Anterior insula**	L	− 28	15	− 19	34	ns	ns	ns	ns	ns	ns	ns	−	+
*Temporal pole*	L					ns	ns	ns	ns	ns	ns	ns	−	+
*Inferior frontal gyrus (p.orbitalis)*	L					ns	ns	ns	ns	ns	ns	ns	−	+
**Middle temporal gyrus**	L	− 60	− 60	13	30	ns	ns	ns	ns	+	−	ns	ns	ns
**Middle frontal gyrus**	L	− 45	52	16	29	ns	ns	ns	ns	+	−	ns	ns	ns
**Supramarginal gyrus**	R	67	− 35	43	29	ns	ns	ns	−	ns	−	ns	ns	ns

Among brain regions showing significant Control × Group interaction, we conducted further analyses to see whether there was a significant condition difference in activity at these brain regions for each group. In Pro and Rea columns, “ + ” and “ − ” separately indicate activation and deactivation; in DIF column, “ + ” indicates greater brain activation or weaker deactivation in proactive vs. reactive conditions and opposite differences are represented by “ − ”. Brain regions in bold are at the peak MNI coordinates of clusters. Other brain regions in these clusters are in italics

*ID* Iron deficiency, *IS* Iron sufficiency, *Hemi* Hemisphere, *R* Right, *L* Left, *Pro* Proactive, *Rea* reactive, *DIF* Differences

### Cue-elicited brain activity

#### Three groups recruited common brain regions to process cues

Compared to the reactive condition, all groups showed greater activity in the proactive condition at the visual cortex, fronto-parietal, and subcortical regions, such as bilateral lingual gyrus, bilateral fusiform gyrus, right middle frontal gyrus, bilateral precentral gyrus, right superior/inferior parietal lobe, bilateral thalamus, and right dorsal striatum (see these brain regions in Fig. 2 and in Additional file 1: Table S5). Additionally, compared to the proactive condition, all groups showed greater activity in the reactive condition at brain regions that are widely distributed, including the bilateral parts of the superior medial gyrus, inferior frontal gyrus, postcentral gyrus, insula and operculum, cingulate cortex, hippocampus, precuneus, and occipital gyrus (see all brain regions in Fig. 2 and Additional file 1: Table S5).

#### Differences between groups in brain regions recruited to process cues

As shown in Table 1 and Fig. 3, the iron-sufficient group showed greater activity in reactive vs. proactive conditions at the left superior frontal gyrus (SFG) and left supplemental motor area (SMA) and showed greater activity in proactive vs. reactive conditions at the left caudate nucleus and left putamen. The postnatal ID group showed greater activity in reactive vs. proactive conditions at the bilateral superior temporal gyrus (STG), bilateral posterior insula, left posterior rolandic operculum (RO), left heschl’s gyrus (HesG), and right parahippocampus. The fetal ID group had greater activity in proactive vs. reactive conditions at the left superior orbital gyrus (SorG) and left putamen.

### Target-elicited brain activity

#### Three groups recruited common brain regions to process targets

All groups showed greater activity at the bilateral insula and operculum, left supramarginal gyrus, left olfactory cortex, left amygdala, and left hippocampus in proactive vs. reactive conditions (see all brain regions in Fig. 2 and in Additional file 1: Table S6). In addition, compared to the proactive condition, all groups showed greater activity in the reactive condition at frontal, occipital-temporal, subcortical, and motor regions, such as the right superior frontal gyrus, right anterior cingulate cortex, as well as the bilateral parts of the inferior frontal gyrus, calcarine gyrus, superior occipital gyrus, lingual gyrus, fusiform gyrus, thalamus, dorsal striatum, and precentral gyrus (see all brain regions in Fig. 2 and Additional file 1: Table S6).

#### Differences between groups in brain regions recruited to process targets

As shown in Table 2 and Fig. 4, the iron-sufficient group showed greater activity in proactive vs. reactive conditions at the fronto-parietal network, temporal regions, limbic system, and insula, such as the orbital part of left inferior frontal gyrus (IFGor), bilateral posterior precuneus, bilateral middle cingulate cortex (MCC), right STG, and left insula (see all brain regions in Table 2). Such condition differences shown by the iron-sufficient group were reversed in the postnatal ID group, which showed greater activity in reactive vs. proactive conditions at widely-distributed brain regions, including the bilateral parts of the middle orbital gyrus (MorG), superior medial gyrus (SmeG), superior parietal lobule (SPL), precuneus, middle and inferior temporal gyrus, as well as left MCC, right middle occipital gyrus, and right caudate nucleus (see all brain regions in Table 2). The fetal ID group showed greater activity in proactive vs. reactive conditions only at the bilateral parts of SorG, MorG, and SmeG.

## Discussion

This study found commonalities and differences in behavioral and neuroimaging results across the 3 groups defined by iron status in early life. Behavioral results indicated that children with or without early ID were able to use proactive and reactive cognitive control according to contextual demands. Due to different demands between proactive and reactive conditions, the cues and targets in these 2 conditions activated different brain regions, some of which were similar across groups. Specifically, all 3 groups showed greater cue-elicited activity in reactive vs. proactive conditions at brain regions from the fronto-parietal, sensorimotor, and cingulo-opercular networks, which are associated with maintaining response rules and monitoring incoming information [41–43]. In contrast, the cues in proactive vs. reactive conditions elicited greater activity in visual regions, such as the fusiform and lingual gyrus [44], associated with processing color information, as well as in the cortical-striatum-thalamus pathway, associated with selecting and updating rule representation [45]. Such brain activity elicited by cues in the proactive condition was not induced until targets arrived in the reactive condition. The targets in the proactive condition induced greater activity than that in the reactive condition in sensorimotor and cingulo-opercular networks associated with selecting and executing responses [46, 47]. In sum, to process the cues and targets in proactive vs. reactive conditions, three groups showed commonality in recruited brain regions.

Such similarities notwithstanding, the 3 groups differed in the activation of some brain regions. For the iron-sufficient group only, the cues in proactive vs. reactive conditions elicited greater activity at left dorsal striatum, associated with selecting or updating rule representation [48], whereas the cues in reactive vs. proactive conditions elicited greater activity at left SFG and SMA, associated with maintaining response rules [47, 49]. Only the iron-sufficient group made faster responses to the targets in proactive vs. reactive conditions by eliciting greater activity at several brain regions within the fronto-parietal and cingulo-opercular networks [43], such as IFGor, SMA, SPL, MCC, and insula. These findings indicate that, compared to the 2 ID groups, the iron-sufficient group recruited more brain resources to make faster responses in proactive vs. reactive conditions. Thus, consistent with animal studies [9, 50, 51], ID in the early life of human beings may impair the development of such brain networks as the fronto-parietal and cingulo-opercular networks. Such impairment in brain development might lead to the observed differences in the brain networks recruited to support cognitive control between pre-adolescent children with early ID and those who were iron-sufficient in infancy.

The postnatal ID group showed greater activity elicited by cues and targets in reactive vs. proactive conditions. Specifically, the cues in the reactive condition activated brain regions related to speech repetition, memory, and linguistic processing [52], such as bilateral posterior insula [53, 54], left RO [55], left HesG [56], bilateral STG [57], and right parahippocampus [58]. These results suggest that the postnatal ID group might have used verbal rehearsal strategies to maintain general task rules in the memory system during the cue period. To process the targets in reactive vs. proactive conditions, the postnatal ID group activated widely-distributed brain regions from the fronto-parietal [25, 42], cingulo-opercular [43, 59], sensorimotor [41], and temporal-occipital [60] networks, associated with processing visual stimuli, integrating multi-sensory information, selecting rules and responses, and making responses. Thus, in contrast to the other two groups, the postnatal ID group appeared to use strategies and extra brain resources to support cognitive control in reactive vs. proactive conditions, suggesting that children with postnatal ID might not be able to use informative cues to stimulate proactive control as well as iron-sufficient children, or the informative cues in the proactive condition might interfere with postnatal ID children’s use of strategies that were internally driven in the reactive condition.

The fetal ID group recruited the orbital gyrus and putamen to process the cues and targets in proactive vs. reactive conditions. These two brain regions are in the brain pathways responsible for processing rewards and monitoring outcomes [61]. Thus, the informative cues in the proactive condition appeared to activate the reward pathway in the fetal ID group. This interpretation is consistent with previous studies showing that early ID is associated with later alterations in sensitivity to reward and punishment [8, 14, 62, 63].

The observed group differences suggest that the alteration of brain networks supporting cognitive control in childhood relates to ID timing in early life. Such findings are consistent with previous studies [29, 30], which detected differences between fetal and postnatal ID in their influences on cognitive and motor development. Such differences related to ID timing might be due to the trajectories of brain development in early life. For example, the brain undergoes rapid development from the fetal to the early postnatal period through neurulation, neuronal proliferation, neural migration, apoptosis, synaptogenesis, and myelination [27]. These neural developmental processes might be differently affected by fetal vs. postnatal ID. Furthermore, due to developmental adaptation and neuroplasticity, children with early ID at different times might have developed different compensatory brain networks or paths to support cognitive control in childhood [64].

## Strengths and limitations

This study could not address how fetal and postnatal ID jointly affect cognitive and brain development as the sample size did not allow us to create a group with ID at both fetal and postnatal periods. Additionally, it would be more informative if neuroimaging data were collected at more time points in addition to the one at 10-year-old. Despite these limitations, this study is based on a longitudinal project that has followed participants for about 10 years. The data are not only rare but also provide important insight into the long-term effects of early ID on brain development.

## Conclusions

Despite the iron supplement in infancy, early ID was associated with altered brain activity during cognitive control about 10 years later. The altered brain functions differed between children with fetal and postnatal ID. These findings support the need to prevent ID during critical/sensitive periods of brain development. This is especially important considering the persistent prevalence of ID in pregnant women and infants [2]. Additionally, the findings suggest that iron treatment alone cannot entirely prevent the long-lasting alterations of brain functions related to early ID. Developing precise intervention strategies for early ID should consider ID timing. Future studies need to investigate whether the altered brain correlates of cognitive control underlie the associations between early ID and long-term impaired development in both socioemotional and cognitive functioning.

## Supplementary Information

**Additional file 1: 1. The protocol of the behavioral task. 2. The computation of family socioeconomic status. 3. The selection of best-fitting models. Table S1.** The demographics of each group. **Table S2.** Iron status at birth, 9 months, 18 months, and 8-to-11 years. **Table S3.** The AIC values of each model. **Table S4.** Behavioral results for each group and condition. **Table S5.** Common brain regions recruited by three groups to process cues in proactive vs. reactive conditions. **Table S6.** Common brain regions recruited by three groups to process targets in proactive vs. reactive conditions.

## Data Availability

The de-identified datasets generated and analyzed in the current study are available at https://doi.org/10.57760/sciencedb.07780.

## Abbreviations

AFNI: Analysis of Functional NeuroImages
AIC: Akaike’s Information Criterion
ANTs: Advanced normalization tools
CI: Confidence interval
FD: Framewise displacement
Hb: Hemoglobin
HesG: Heschl’s gyrus
ID: Iron deficiency
IFGor: Inferior frontal gyrus (p. orbitalis)
MCC: Middle cingulate cortex
MCV: Mean corpuscular volume
MNI: Montreal neurological institute
MorG: Mid orbital gyrus
RDW: Red cell distribution width
RO: Rolandic operculum
SF: Serum ferritin
SFG: Superior frontal gyrus
SMA: Supplemental motor area
SmeG: Superior medial gyrus
SorG: Superior orbital gyrus
SPL: Superior parietal lobe
SPM: Statistical parametric mapping
STG: Superior temporal gyrus
TE: Echo time
TR: Repetition time
ZPP/H: Zinc protoporphyrin/heme ratio
