# Supplementary material for: Differentiation between fetal and postnatal iron deficiency in altering brain substrates of cognitive control in pre-adolescence
Source: BMC Med. 2023 May 4;21:167. doi: 10.1186/s12916-023-02850-6 (PMC10161450; doi:10.1186/s12916-023-02850-6)
Supplement: Supplementary file 1 — Additional file 1: 1. The protocol of the behavioral task. 2. The computation of family socioeconomic status. 3. The selection of best-fitting models. Table S1. The demographics of each group. Table S2. Iron status at birth, 9 months, 18 months, and 8-to-11 years. Table S3. The AIC values of each model. Table S4. Behavioral results for each group and condition. Table S5. Common brain regions recruited by three groups to process cues in proactive vs. reactive conditions. Table S6. Common brain regions recruited by three groups to process targets in proactive vs. reactive conditions. [file 12916_2023_2850_MOESM1_ESM.docx]

**Supplementary Online Content**

1. The protocol of the behavioral task
2. The computation of family socioeconomic status
3. The selection of best-fitting models

**Table S1.** The demographics of each group

**Table S2.** Iron status at birth, 9 months, 18 months, and 8-to-11 years

**Table S3.** The AIC values of each model

**Table S4.** Behavioral results for each group and condition

**Table S5.** Common brain regions recruited by three groups to process cues in proactive vs. reactive conditions

**Table S6.** Common brain regions recruited by three groups to process targets in proactive vs. reactive conditions

1. **The protocol of the behavioral task**

***Step 1.*** *Preparation*

The version of the behavioral task was selected.

***Step 2.*** *Rule selection*

The experimenter explained the rules to the children, “This game has the Winnie and Donald sessions. *First*, let’s play the Winnie session, in which green or red borders would present either before the onset of targets or simultaneously with targets. No matter when the colored borders are presented, the response rules are consistent. When the border is red, the response rule is to judge whether the target is a food picture. In contrast, when the border is green, the response rule is to judge whether the target is an animal picture. You always press the LEFT key when the answer is YES and the RIGHT key when the answer is NO.”

After ensuring that participants understood the rules, the experimenter continued explaining the rule of the Donald session. The experimenter said, “When it is Donald session, the rule is reversed compared to the Winnie session. When the border is red, the rule is to judge whether the target is an animal picture. In contrast, when the border is green, the rule is to judge whether the target is a food picture. You always press the LEFT key when the answer is YES and the RIGHT key when the answer is NO.”

***Step 3.*** *Practice and pre-test*

After explaining the rules, participants started practicing. If necessary, the experimenter further explained the rules. After practicing, the children performed the pre-test. Only when the accuracy reached 90% could children start preparing for going into the MRI scanner.

***Step 4.*** *Formal test*

Children performed formal tasks in the MRI scanner.

1. **The computation of family socioeconomic status**

The information on household income, parental education and occupation was collected by qualified project personnel when the cohort study was set up (i.e., at 6-week-old and at 9-month-old). According to the method used in a previous study^[[1]](#footnote-1)^, we categorized parental education, parental occupation, and household income into several levels and assigned a score to each level (Additional file 1: Table S1). Specifically, annual household income was categorized into low (< ¥10,000; score 1), medium-low (¥10,000-29,999, score 2), medium (¥30,000-49,999, score 3), medium-high (¥50,000-99,999, score 4), and high (≥ ¥100,000, score 5). Parental education was categorized into low (lower than high school, score 1), medium (high school graduate or equivalent, score 2), and high (college graduate or above, score 3). Parental occupation was categorized into low (manual worker, farmer, or unemployed; score 1), medium (businessman or clerk; score 2), and high (professional, manager, or government employee, score 3). The scores of parental education, parental occupation, and household income were combined to represent the general socioeconomic status of families. According to such general indicator, we categorized families into low (scores 5–7), middle (scores 8–10), and high (scores 11–17) levels, shown in Table S1. We carried out statistical analyses (i.e., Fisher’s exact test) to test whether there were significant differences between the three groups in the distribution of participants. The results indicated that there was no significant difference between groups in parental education (fathers: χ^2^ = 2.1, *p* = 0.7; mothers: χ^2^ = 7.2, *p* = 0.1), parental occupation (fathers: χ^2^ = 1.7, *p* = 0.8; mothers: χ^2^ =5.4, *p* = 0.2), household income (χ^2^ = 8.6, *p* = 0.3), and general social economic status (χ^2^ = 2.2, *p* = 0.7).

1. **The selection of best-fitting models**

For accuracy, the best fitting model included only main effects (i.e., Group, Control, and Switch). For reaction times, the best fitting model included main effects and all interactions (i.e., Group × Control; Control × Switch; Group × Switch; Group × Control × Switch). The AIC values of all candidate models were listed in Table S3.

**Table S1. The demographics of each group**

|  | **Fetal ID** | **Postnatal ID** | **IS** |
| --- | --- | --- | --- |
| **Sample size, *n*** | 20 | 24 | 27 |
| **Age, M (SD), year** | 9.9 (1.0) | 9.9 (0.8) | 10.2 (0.9) |
| **Sex** |  |  |  |
| Boys, *n* (%) | 10 (50) | 14 (58) | 13 (48) |
| Girls, *n* (%) | 10 (50) | 10 (42) | 14 (52) |
| **Head Motion, M (SD), mm** | 0.3 (0.1) | 0.3 (0.1) | 0.3 (0.1) |
| **Parental age (at birth)** |  |  |  |
| Mother, M (SD), year | 26.5 (3.3) | 27.3 (4.2) | 26.8 (4.6) |
| Father, M (SD), year | 28.4 (3.7) | 29.5 (4.4) | 29.5 (4.2) |
| **Maternal education** |  |  |  |
| Low, *n* (%) | 5 (27.8) | 9 (42.9) | 10 (40.0) |
| Medium, *n* (%) | 2 (11.1) | 6 (28.6) | 9 (36.0) |
| High, *n* (%) | 11 (61.1) | 6 (28.6) | 6 (24.0) |
| **Paternal education** |  |  |  |
| Low, *n* (%) | 5 (27.8) | 6 (28.6) | 9 (36.0) |
| Medium, *n* (%) | 4 (22.2) | 8 (38.1) | 8 (32.0) |
| High, *n* (%) | 9 (50.0) | 7 (33.3) | 8 (32.0) |
| **Maternal occupation** |  |  |  |
| Low, *n* (%) | 8 (44.4) | 13 (61.9) | 18 (72.0) |
| Medium, *n* (%) | 9 (50.0) | 5 (23.8) | 6 (24.0) |
| High, *n* (%) | 1 (5.6) | 3 (14.3) | 1 (4.0) |
| **Paternal occupation** |  |  |  |
| Low, *n* (%) | 3 (16.7) | 6 (28.6) | 5 (20.0) |
| Medium, *n* (%) | 11 (61.1) | 12 (57.1) | 17 (68.0) |
| High, *n* (%) | 4 (22.2) | 3 (14.3) | 3 (12.0) |
| **Household income** |  |  |  |
| Low, *n* (%) | 0 (0) | 1 (5.0) | 1 (4.0) |
| Medium-low, *n* (%) | 3 (17.6) | 1 (5.0) | 5 (20.0) |
| Medium, *n* (%) | 3 (17.6) | 4 (20.0) | 7 (28.0) |
| Medium-high, *n* (%) | 5 (29.4) | 5 (25.0) | 9 (36.0) |
| High, *n* (%) | 6 (35.3) | 9 (45.0) | 3 (12.0) |
| **Socioeconomic status** |  |  |  |
| Low, *n* (%) | 2 (11.1) | 2 (10.0) | 4 (16.0) |
| Middle, *n* (%) | 4 (22.2) | 8 (40.0) | 9 (36.0) |
| High, *n* (%) | 12 (66.7) | 10 (50.0) | 12 (48.0) |

Abbreviations: ID, iron deficiency; IS, iron sufficiency.

**Table S2. Iron status at birth, 9 months, 18 months, and 8-to-11 years**

| **Iron measures^a^** | **Fetal ID** | **Postnatal ID** | **IS** |
| --- | --- | --- | --- |
| **Newborn** |  |  |  |
| Hb, *M (SD)*, g/L | 147.3 (21.4) | 148.5 (18.3) | 147.2 (16.2) |
| SF, *M (SD)*, µg/L | 110.3 (74.5) | 207.8 (84.6) | 195.5 (51.9) |
| ZPP/H, *M (SD)*, µmol/mol | 127.7 (28.7) | 97.1 (14.9) | 90.7 (17.3) |
| **9 months** |  |  |  |
| Hb, *M (SD)*, g/L | 108.1 (11.7) | 108.7 (8.6)^b^ | 116.2 (7.9)^c^ |
| SF, *M (SD)*, µg/L | 31.7 (27.6) | 31.1 (33.9) | 57.9 (36.8) |
| ZPP/H, *M (SD)*, µmol/mol | 143.0 (123.7) | 136.9 (47.5) | 94.8 (25.9) |
| MCV, *M (SD)*, fl | 76.3 (6.4) | 73.0 (3.6) | 79.9 (3.0) |
| RDW, *M (SD)*, % | 13.6 (1.9) | 14.6 (1.8) | 12.7 (0.7) |
| **18 months** |  |  |  |
| Hb, *M (SD)*, g/L | 125.5 (7.6) | 119.9 (8.0) | 121.3 (6.9) |
| SF, *M (SD)*, ng/mL | 81.9 (25.0) | 70.6 (31.7) | 86.9 (24.0) |
| ZPP/H, *M (SD)*, µmol/mol | 53.0 (45.5) | 38.7 (23.1) | 47.2 (13.9) |
| MCV, *M (SD)*, fl | 80.9 (2.3) | 80.8 (3.4) | 82.3 (2.5) |
| RDW, *M (SD)*, % | 12.4 (0.7) | 12.8 (0.8) | 12.3 (0.8) |
| **8-11 years** |  |  |  |
| Hb, *M (SD)*, g/L | 134.9 (9.6) | 130.3 (10.9) | 137.5 (8.4) |
| SF, *M (SD)*, µg/L | 45.3 (29.0) | 38.7 (19.1) | 40.9 (24.3) |
| MCV, *M (SD)*, fl | 82.3 (14.8) | 84.9 (3.8) | 86.9 (2.4) |
| RDW, *M (SD)*, % | 12.6 (0.7) | 12.9 (0.9) | 12.4 (0.5) |

Abbreviations: ID, iron deficiency; IS, iron sufficiency; Hb, Hemoglobin; SF, Serum ferritin; ZPP/H, Zinc protoporphyrin/heme ratio; MCV, Mean corpuscular volume; RDW, Red cell distribution width.

^a^ MCV and RDW were not measured at birth; ZPP/H was not measured at 8-11 years

^b^ There were 1 child with Hb between 90-100 g/L, 16 children with Hb between 100-110 g/L and 7 children with Hb > 110g/L.

^c^ There were 7 children with Hb between 100 -110 g/L and 20 children with Hb > 110g/L.

**Table S3. The AIC values of each model**

| **Behavioral Measures** | **Model 1^a^** | **Model 2^b^** | **Model 3^c^** |
| --- | --- | --- | --- |
| **Accuracy** | -605.9 | -615.3 | -645.2 |
| **Reaction Times** | 3436.6 | 3457.2 | 3500.5 |

Abbreviations: AIC, Akaike Information Criterion.

^a^ Model 1 includes all main effects (i.e., Group, Control, and Switch) and interactions (i.e., Group × Control; Control × Switch; Group × Switch; Group × Control × Switch)

^b^ Model 2 includes all main effects (i.e., Group, Control, and Switch) and 2-level interactions (i.e., Group × Control; Control × Switch; Group × Switch)

^c^ Model 3 includes only main effects (i.e., Group, Control, and Switch)

**Table S4. Behavioral results for each group and condition**

| **Measures** | **Control** | **Group** | | |
| --- | --- | --- | --- | --- |
|  |  | **Fetal ID** | **Postnatal ID** | **IS** |
| **Reaction times,**  ***M (SD)*, ms** | Proactive | 1289.7 (169.9) | 1254.8 (238.4) | 1174.8 (209.4) |
|  | Reactive | 1705.2 (198.9) | 1690.3 (291.8) | 1613.3 (280.0) |
| **Accuracy, *M(SD)*** | Proactive | 0.84 (0.10) | 0.89 (0.08) | 0.91 (0.07) |
|  | Reactive | 0.85 (0.10) | 0.88 (0.09) | 0.91 (0.06) |

*Note.* There was no group difference in reaction times and all groups responded faster in proactive vs. reactive conditions. The fetal ID group was lower in overall accuracy than both the postnatal ID and iron-sufficient groups. Abbreviations: ID, iron deficiency; IS, iron sufficiency.

**Table S5. Common brain regions recruited by three groups to process cues in proactive vs. reactive conditions**

| **Cluster** | **Hemi** | **Peak MNI coordinate** | | | **Size** | **Pro** | **Rea** | **DIF** |
| --- | --- | --- | --- | --- | --- | --- | --- | --- |
|  |  | **x** | **y** | **z** |  |  |  |  |
| **Insula** | L | -40 | -8 | 1 | 1477 | - | + | - |
| *Rolandic operculum* | L |  |  |  |  | - | ns | - |
| *Heschl’s gyrus* | L |  |  |  |  | ns | + | - |
| *Inferior frontal gyrus*  *(p.orbitalis)* | L |  |  |  |  | ns | + | - |
| *Inferior frontal gyrus*  *(p.triangularis)* | L |  |  |  |  | ns | + | - |
| **Inferior frontal gyrus**  **(p.orbitalis)** | R | 50 | 30 | -4 | 1189 | - | + | - |
| *Inferior frontal gyrus*  *(p.opercularis)* | R |  |  |  |  | ns | + | - |
| *Anterior rolandic operculum* | R |  |  |  |  | - | + | - |
| *Anterior insula* | R |  |  |  |  | - | + | - |
| **Fusiform gyrus** | R | 30 | -65 | -7 | 638 | ns | - | + |
| *Lingual gyrus* | R |  |  |  |  | + | - | + |
| *Calcarine gyrus* | R |  |  |  |  | ns | - | + |
| **Lingual gyrus** | L | -20 | -78 | -4 | 507 | + | + | + |
| *Fusiform gyrus* | L |  |  |  |  | ns | - | + |
| **Precentral gyrus** | L | -28 | -20 | 66 | 471 | - | - | - |
| *Postcentral gyrus* | L |  |  |  |  | - | ns | - |
| **Middle occipital gyrus** | L | -30 | -100 | 1 | 463 | - | - | - |
| *Calcarine gyrus* | L |  |  |  |  | - | ns | - |
| **Middle occipital gyrus** | R | 27 | -98 | 3 | 412 | - | ns | - |
| *Inferior occipital gyrus* | R |  |  |  |  | - | - | - |
| *Calcarine gyrus* | R |  |  |  |  | - | ns | - |
| **Hippocampus** | L | -28 | -35 | -7 | 290 | - | + | - |
| *Parahippocampus* | L |  |  |  |  | - | + | - |
| **Precentral gyrus** | L | -38 | -3 | 53 | 286 | ns | - | + |
| **Putamen** | L | -20 | 2 | 6 | 171 | ns | - | + |
| *Caudate nucleus* | L |  |  |  |  | ns | - | + |
| **Precentral gyrus** | R | 47 | 2 | 36 | 148 | + | - | + |
| *Middle frontal gyrus* | R |  |  |  |  | ns | - | + |
| **Superior medial gyrus** | B | 0 | 37 | 36 | 140 | ns | + | - |
| **Inferior occipital gyrus** | L | -43 | -75 | -17 | 138 | - | - | - |
| **Hippocampus** | R | 30 | -25 | -9 | 123 | - | + | - |
| **Supplementary motor area** | L | -5 | 2 | 68 | 89 | + | - | + |
| **Precuneus** | B | 2 | -48 | 76 | 89 | - | ns | - |
| **Inferior parietal lobule** | R | 32 | -53 | 53 | 88 | + | ns | + |
| *Superior parietal lobule* | R |  |  |  |  | ns | - | + |
| **Postcentral gyrus** | R | 67 | -15 | 31 | 82 | ns | + | - |
| **Caudate nucleus** | R | 20 | 12 | 11 | 75 | ns | - | + |
| *Putamen* | R |  |  |  |  | ns | - | + |
| *Pallidum* | R |  |  |  |  | - | - | + |
| **Superior frontal gyrus** | L | -18 | -5 | 76 | 59 | - | ns | - |
| **Superior occipital gyrus** | L | -10 | -95 | 13 | 52 | + | + | + |
| *Cuneus* | L |  |  |  |  | + | ns | + |
| **Cuneus** | R | 7 | -85 | 38 | 52 | ns | + | - |
| *Cuneus* | L |  |  |  |  | - | ns | - |
| **Supplementary motor area** | B | -3 | -8 | 56 | 41 | - | - | - |
| **Middle temporal gyrus** | R | 55 | -33 | -14 | 40 | ns | + | - |
| **Middle frontal gyrus** | L | -43 | 47 | 31 | 38 | ns | + | - |
| **Thalamus** | L | -8 | -18 | 3 | 34 | ns | - | + |
| **Fusiform gyrus** | R | 32 | -33 | -24 | 34 | - | - | - |
| **Supplementary motor area** | R | 5 | 15 | 71 | 33 | ns | + | - |
| **Angular gyrus** | L | -38 | -60 | 26 | 32 | - | ns | - |
| **Supramarginal gyrus** | R | 67 | -30 | 36 | 31 | - | ns | - |
| **Middle cingulate cortex** | B | 2 | 0 | 43 | 30 | - | - | - |

*Note*. Common brain regions were recruited by the three groups to process cues in proactive vs. reactive conditions. In the Pro and Rea columns, “+” and “-” separately indicate activation and deactivation; in the DIF column, “+” indicates greater brain activation or weaker deactivation in proactive vs. reactive conditions and the opposite difference is represented by “-”. Brain regions in bold are at the peak MNI coordinates of clusters. Other brain regions within these clusters were presented as italics. Hemi, hemisphere; R, right; L, left; B, bilateral; Pro, proactive; Rea, reactive; DIF, differences.

**Table S6. Common brain regions recruited by three groups to process targets in proactive vs. reactive conditions**

| **Cluster** | **Hemi** | **Peak MNI coordinate** | | | **Size** | **Pro** | **Rea** | **DIF** |
| --- | --- | --- | --- | --- | --- | --- | --- | --- |
|  |  | **x** | **y** | **z** |  |  |  |  |
| **Fusiform gyrus** | B | -25 | -73 | -4 | 6738 | + | + | - |
| *Lingual gyrus* | B |  |  |  |  | - | + | - |
| *Superior occipital gyrus* | B |  |  |  |  | - | + | - |
| *Middle occipital gyrus* | B |  |  |  |  | + | + | - |
| *Cuneus* | B |  |  |  |  | - | + | - |
| *Precuneus* | B |  |  |  |  | - | + | - |
| *Superior parietal lobule* | B |  |  |  |  | ns | + | - |
| *Calcarine gyrus* | B |  |  |  |  | - | + | - |
| **Precentral gyrus** | L | -43 | -3 | 43 | 1208 | + | + | - |
| *Postcentral gyrus* | L |  |  |  |  | - | + | - |
| *Inferior frontal gyrus*  *(p.opercularis)* | L |  |  |  |  | + | + | - |
| *Inferior frontal gyrus*  *(p. Triangularis)* | L |  |  |  |  | + | + | - |
| **Precentral gyrus** | R | 45 | 2 | 31 | 1140 | - | + | - |
| *Middle frontal gyrus* | R |  |  |  |  | ns | + | - |
| *Inferior frontal gyrus*  *(p.opercularis)* | R |  |  |  |  | ns | + | - |
| *Inferior frontal gyrus*  *(p. Triangularis)* | R |  |  |  |  | ns | + | - |
| **Anterior rolandic operculum** | L | -43 | -3 | 11 | 302 | + | + | + |
| *Central insula* | L |  |  |  |  | + | + | + |
| **Postcentral gyrus** | B | 17 | -38 | 73 | 262 | ns | + | - |
| *Paracentral lobe* | B |  |  |  |  | ns | + | - |
| *Supplementary motor area* | R |  |  |  |  | ns | + | - |
| **Putamen** | L | -18 | 5 | 8 | 226 | + | + | - |
| *Caudate nucleus* | L |  |  |  |  | ns | + | - |
| **Insula** | R | 37 | 0 | 13 | 181 | + | + | + |
| *Rolandic operculum* | R |  |  |  |  | + | + | + |
| *Putamen* | R |  |  |  |  | + | + | + |
| **Putamen** | R | 20 | 10 | 6 | 144 | + | + | - |
| *Caudate nucleus* | R |  |  |  |  | ns | + | - |
| **Thalamus** | L | -5 | -25 | -2 | 126 | ns | + | - |
| **Superior frontal gyrus** | R | 20 | 67 | 16 | 124 | - | ns | - |
| *Superior medial gyrus* | R |  |  |  |  | - | ns | - |
| **Thalamus** | R | 7 | -15 | 3 | 118 | ns | + | - |
| **Middle orbital gyrus** | B | 0 | 47 | -12 | 76 | - | ns | - |
| *Anterior cingulate cortex* | B |  |  |  |  | - | ns | - |
| **Superior medial gyrus** | L | 0 | 50 | 41 | 55 | - | ns | - |
| **Hippocampus** | L | -18 | -8 | -17 | 54 | + | + | + |
| *Amygdala* | L |  |  |  |  | + | ns | + |
| *Olfactory cortex* | L |  |  |  |  | ns | - | + |
| **Superior temporal gyrus** | R | 72 | -23 | 1 | 54 | - | ns | - |
| **Inferior temporal gyrus** | R | 57 | -8 | -29 | 40 | ns | + | - |
| **Postcentral gyrus** | L | -53 | -20 | 21 | 36 | + | ns | + |
| *Supramarginal gyrus* | L |  |  |  |  | + | + | + |
| **Supplementary motor area** | L | -5 | 15 | 61 | 34 | + | + | - |
| **Middle orbital gyrus** | R | 7 | 62 | -7 | 33 | ns | - | + |
| **Middle temporal gyrus** | L | -43 | -50 | 18 | 30 | - | ns | - |
| **Inferior temporal gyrus** | L | -55 | -33 | -19 | 29 | ns | + | - |
| **Insula** | R | 32 | 15 | -14 | 27 | ns | - | + |
| **Superior temporal gyrus** | R | 70 | -38 | 18 | 27 | - | ns | - |
| **Supplementary motor area** | R | 12 | 7 | 63 | 27 | + | + | + |

*Note*. Common brain regions were recruited by three groups to process targets in proactive vs. reactive conditions. In Pro and Rea columns, “+” and “-” separately indicate activation and deactivation; in DIF column, “+” indicates greater brain activation or weaker deactivation in proactive vs. reactive conditions and the opposite difference is represented by “-”. Brain regions in bold are at the peak MNI coordinates of clusters. Other brain regions within these clusters were presented as italic. Hemi, hemisphere; R, right; L, left; B, bilateral; Pro, proactive; Rea, reactive; DIF, differences.

1. Xiang L, Su Z, Liu Y, Zhang X, Li S, Hu S, Zhang H. Effect of family socioeconomic status on the prognosis of complex congenital heart disease in children: an observational cohort study from China. Lancet Child Adolesc Health 2018, 2(6):430-439. [↑](#footnote-ref-1)
